# Hemotropic *Mycoplasma* and *Bartonella* Species Diversity in Free-Roaming Canine and Feline from Luanda, Angola

**DOI:** 10.3390/pathogens10060735

**Published:** 2021-06-10

**Authors:** João R. Mesquita, Ana C. Oliveira, Frederico Neves, Jose R. Mendoza, Maria F. Luz, Inês Crespo, Thays F. dos Santos, Sérgio Santos-Silva, Hugo Vilhena, Patrícia F. Barradas

**Affiliations:** 1 Institute of Biomedical Sciences Abel Salazar (ICBAS), University of Porto, 4050-313 Porto, Portugal; jrmesquita@icbas.up.pt; 2 Epidemiology Research Unit (EPIUnit), Instituto de Saúde Pública da Universidade do Porto, Rua das Taipas 135, 4050-091 Porto, Portugal; 3 Casa dos Animais Veterinary Clinic, Luanda, Angola; olivecris@hotmail.com (A.C.O.); joseriveromendoza@gmail.com (J.R.M.); franciscazarco@gmail.com (M.F.L.); 4 Department of Veterinary Sciences, Vasco da Gama Universitary School (EUVG), 3020-210 Coimbra, Portugal; fredericomiguelneves@gmail.com; 5 Center for Investigation Vasco da Gama (CIVG), Department of Veterinary Sciences, Vasco da Gama University School (EUVG), 3020-210 Coimbra, Portugal; ines.r.crespo@gmail.com (I.C.); hcrvilhena@hotmail.com (H.V.); 6 Department of Epidemiology and Public Health, Universidade Federal Rural do Rio de Janeiro, BR-465, Km 7, Seropédica, RJ 23897-000, Brazil; thaysfigueiroa@outlook.com.br; 7 Polytechnic Institute of Coimbra (IPC), Agrarian School, 3045-093 Coimbra, Portugal; sergiosilva.1999@hotmail.com; 8 Animal and Veterinary Research Center (CECAV), University of Trás-os-Montes and Alto Douro (UTAD), 5000-801 Vila Real, Portugal; 9 University Veterinary Hospital of Coimbra, 3020-210 Coimbra, Portugal; 10 Polytechnic Institute of Viana do Castelo (IPVC), Agrarian School, 4990-706 Ponte de Lima, Portugal

**Keywords:** hemoplasma, *Bartonella henselae*, free-roaming, Angola

## Abstract

Free-roaming dogs and cats represent potential reservoirs for zoonotic vector-borne pathogens shedding to the human population. Given the health impact of these pathogens, we searched free-roaming dogs and cats included in an animal population control program from Luanda, Angola, for *Bartonella* and hemotropic mycoplasma infection. We report the detection of *Bartonella henselae* (2/66; 3%), *Candidatus* Mycoplasma haemominutum (5/66; 7.5%) and *Mycoplasma haemofelis* (1/66; 1.5%) in cats. One dog was found positive for *Mycoplasma haemocanis* (1/20; 5%). This is the first report of *Bartonella henselae* infections in stray cats and of hemotropic mycoplasmas in cats and dogs from Angola. Despite the relatively small sample size, our results sustain the hypothesis of uncontrolled circulation of these agents in highly mobile synanthropic animal populations of Luanda. Population and vector control could contribute to reducing the likelihood for animal-to-animal and animal-to-human transmission.

## 1. Introduction

Many vector-borne organisms are considered emerging or re-emerging pathogens, with increasing comparative biomedical importance worldwide [1]. People and animals share many of these microorganisms and diseases, with circa 60 percent of human infections estimated to have an animal origin [2], many of which are transmitted by arthropod vectors [3]. Domestic and peri-domestic animals represent a bridge for the emergence of human diseases. Free-roaming animals, including dogs and cats, are typically not under human control and are hence not submitted to vaccination or ectoparasitic control, being considered as potential reservoirs for zoonotic vector-borne pathogens [4].

*Bartonella* spp. are a worldwide distributed Gram-negative, hemotropic, and rod-shaped Alphaproteobacteria [5], mainly transmitted by arthropod vectors [6]. Elements from this genus are fastidious, slow-growing, and facultative intracellular bacteria, highly adapted to a broad spectrum of mammalian reservoir hosts [7,8,9]. *Bartonella henselae* and *B. clarridgeiae* are known to be the agents of cat-scratch disease (CSD) in humans, with cats being recognized as a reservoir for both species [10]. However, unlike humans, cats infected with *Bartonella* do not usually develop any symptoms but present relapsing bacteraemia for months or years [11]. The dog may also be a host for *B. henselae* and is considered the primary reservoir for *B. vinsonii berkhoffii*, causing endocarditis in both dogs and humans [12]. Blood-sucking arthropod vectors (fleas, lice, sand flies, biting flies, and ticks) ingest intra-erythrocytic *Bartonella* spp. during the blood meal, after which transmission to animals and humans can occur mainly by inoculation of *Bartonella*-contaminated arthropod feces via animal scratches or bites or host self-inflected contamination of wounds, or by bites of infected vectors [13]. Vector bites are a well-documented mode of transmission, as demonstrated by *Lutzomyia verrucarum* sand flies, a vector of *B. bacilliformis* [14]. Moreover, the detection of *B. henselae* in questing ticks [15] and the transmission of *B. henselae* between cats through *Ctenocephalides felis* fleas have been described [16].

Various epidemiological studies have been conducted in cats and dogs in many countries, with *Bartonella* DNA detection rates varying greatly. Positive animals were found in several countries such as in Albania (0.7% positive) [17], South Africa (23.5% positive) [18], Italy (from 2.5% to 83.5%) [19,20], Thailand (1.61%) [21], Brazil (24.7%) [22], and China (3.94%) [23]. Detection rates are highly variable in relation to the different geographic areas and studied populations and typically higher mostly where environmental conditions and human behavioral factors are favorable for the survival of their vectors [24]. To the best of our knowledge, only one epidemiologic molecular study evaluated *Bartonella* spp. infection in felines from Angola, showing 1% occurrence in indoor cats [7].

Hemotropic mycoplasmas are small, unculturable, cell wall–deficient, Gram-negative bacteria [25] that adhere to the host’s erythrocytes of numerous domestic and wild animals, such as cats, dogs, rodents, swine, cattle, sheep, bears, and bats [25,26,27]. These bacteria cause diseases that range from asymptomatic infections to acute hemolytic anemia [28]. Few studies on molecular characterization of human *Mycoplasma* infection have been reported so far [29,30], and an increasing body of knowledge has shown pathologic effect when predisposing conditions are present, such as immunodeficiency [30,31]. Several *Mycoplasma* species have been described as infecting wild and domestic animals all over the world. Three species are recognized as affecting cats, *Mycoplasma haemofelis* (Mhf), *Candidatus* Mycoplasma haemominutum (CMhm) and *Candidatus* Mycoplasma turicensis (CMt) [32], and two affecting dogs, *Mycoplasma haemocanis* (Mhc) and *Candidatus* Mycoplasma haematoparvum (CMhp) [33]. However, “CMhm” and “CMt” were already detected in dogs [34,35], and “Candidatus Mycoplasma haematoparvum-like” was found in cats [36]. Transmission of hemotropic mycoplasma species remains cryptic, however, several modes have been suggested. DNA of the agents was already amplified from *C. felis* fleas and *Ixodes* spp. ticks, suggesting a possible vector association [37,38].

On the other hand, infection is still prevalent in some regions where flea infestation is uncommon, and a recent study has also shown that haemoplasmas were not transmitted by *Aedes* mosquitoes [37,39].

Several studies reported on epidemiological aspects of hemotropic *Mycoplasmas* in both cats and dogs from several countries with changing DNA occurrences namely Portugal (CMhm: 41.56%; Mhf: 12.81%; CMt: 1.25%; CMhp: 4.38%) [36]; Albania (CMhm: 21.9%; Mhf: 10.3%; CMt: 5.5%) [17], Spain (CMhm: 9.9%; Mhf: 3.7%; CMt: 0.5%) [40], Italy (CMhm: 12.3%; Mhf: 9.4%; CMt: 4.8%) [41], Romania (CMhm: 72.7%; Mhf: 27.3%) [42], South Africa (CMhm: 38%; Mhf: 15%; CMt: 26%) [43], and Spain (Mhc: 14.3%; CMhp: 0.6%) [44], Italy (Mhc: 4.7%; CMhp: 1.4%) [41], Chile (Mhc: 11.9%; CMhp: 12.2%) [35], and Nigeria (Mhc: 6%) [45], respectively.

Although at least thirteen *Bartonella* species or subspecies have been recognized as agents of human disease, the zoonotic potential of hemotropic *Mycoplasmas* is not yet fully clarified. However, these organisms have occasionally been reported in humans, including anemic patients with acquired immunodeficiency syndrome and systemic lupus erythematosus [46,47,48,49]. Moreover, co-infection with Mhf and *B. henselae* was also diagnosed in an immunodeficiency virus-infected individual from Brazil, suggesting a role of Mhf in human disease and raising alerts for caution when handling blood or tissues from infected animals [31].

Given the veterinary and public health impact of these pathogens, this study aimed to evaluate the species of *Bartonella* and hemotropic hemoplasmas infecting free-roaming dogs and cats from Luanda, Angola, by molecular methods. To our knowledge, this is the first study reporting the occurrence of these infectious agents in free-roaming dogs and cats from Angola.

## 2. Results

All cats and dogs included in this study were free-roaming and crossbreed. The estimated cats’ age ranged from five months to seven years, of which half (33/66; 50%) were males and half were females. The estimated dogs’ age ranged from one to 10 years, of which 58% (11/19) were males. Out of the 66 cat blood specimens tested, two adult animals, one male and one female, (2/66; 3.0%; 95% Confidence Interval (CI): 3.7–10.5) showed to be *Bartonella*-positive for 16S-23S rDNA ITS, while none of the dog blood specimens tested positive for *Bartonella* infection. Sequence analysis of the *Bartonella* 16S–23S rDNA ITS positive amplicons from free-roaming cats from Luanda, Angola, revealed 100% nucleotide identity with *B. henselae* (GenBank accession number MT095053).

Regarding hemotropic hemoplasmas, a total of seven out of 85 free-roaming cats and dogs (8.2%, 95% CI 3.4–16.2) tested positive for PCR amplification of the 16S rRNA gene.

Of the 66 cats tested, six (6/66; 9.1%; 95% CI: 3.4–18.7), two kittens and four adults, showed to be hemoplasma positive, namely five males and one female. Two species were identified: CMhm (5/66; 7.5%) was the dominant species in cats, followed by Mhf (1/66; 1.5%). Sequencing of PCR products was successfully performed in all positive samples. Sequence identity analysis revealed that the five CMhm showed 99.84% and 99.83% similarity with CMhm from Southern Italy (GenBank accession number KR905449) and northern Italy (GenBank accession number EU839985), respectively. Sequence identity analysis of the Mhf derived from the present study presented 99.66% homology with CMhf (GenBank accession number KR905465) from Italy.

Out of the 19 dogs tested, one adult male dog (1/19; 5%; 95% CI: 1.3–24.9) was shown to be Mhc positive. Sequence identity analysis of the Mhc derived from the present study presented 99.42% homology with CMhc (GenBank accession number MT345534) from South Korea.

GenBank accession numbers of *Bartonella* sequences obtained in this study are: MW477483-MW477484 16S-23S rDNA gene of *B. henselae*). GenBank accession numbers of *Mycoplasma* sequences obtained in this study are: MW598399 to MW598403 (16S rRNA gene fragment of CMhm), MW633343 (16S rRNA gene fragment of CMhf), and MW633326 (16S rRNA gene fragment of CMhc).

## 3. Discussion

In the present study, molecular techniques were employed to survey free-roaming Luandan cats and dogs’ blood for *Bartonella* spp. and hemotropic mycoplasmas. Among the 85 tested free-roaming animals, two cats showed to be *B*. *henselae* positive. This result is in accordance with a previous study performed on owned cats from the same city [7]. In contrast, a higher percentage of feline *Bartonella* infections was reported in previous studies performed in other countries from Africa, namely South Africa (8/56; 14%) [18], in Algeria (36/211; 17%) [50], or Zimbabwe (2/25, 8%) [51]. None of the dogs tested positive for this bacterium, a result that is in contrast with former studies carried out in dogs from Tunisia (22/149; 15%) [52] and in Algeria (6/80; 6.25%) [52], but in agreement with studies achieved in rural dogs from Uganda [53] and domestic dogs from Zambia [54].

Evidence for the presence of three hemoplasma species in both free-roaming Luandan dogs and cats was also demonstrated in this study. Mhf, CMhm, and CMt are considered the hemoplasmas species of cats. These species present different pathogenic potential, and comorbidities may influence the disease severity [32]. In dogs, Mhc and CMhp species have been reported, being primarily found in immunocompromised dogs [55]. Out of the 66 free-roaming cats tested, 9% were hemoplasma-positive, with 7.5% CMhm and 1.5% Mhf-positive. The overall occurrence of cat hemoplasma species infection reported in this study was lower when compared to previous studies carried out in South Africa (CMhm: 21.6%; Mhf: 3.9%) [18] but is similar to the occurrence found by our group in a previous study with a group of client-owned indoor cats from the same region, which found four out of 67 cats (6%) being positive for CMhm DNA (data not published). Regarding dogs’ hemoplasmas, the unique species amplified was Mhc, in a single dog. Studies reporting the presence of Mhc in dogs were made in other countries such as Sudan (Mhc: 0.07%) [56], Chile (Mhc: 11.9%) [35], and Korea (Mhc: 38.2%) [57]. It was also interesting to verify that Luandan dogs appeared infected only with Mhc, which is in disagreement with several previous studies [33,35,57]. Nonetheless, we alert caution when interpreting our results given the small sample size and, in some instances, also the scarce detection rate,

This is the first report of *Bartonella* and hemotropic mycoplasma infections diagnosed by PCR in stray dogs and cats from Angola. A number of *Bartonella* spp. are today recognized agents of human disease, and the body of knowledge sustaining the zoonotic potential of hemotropic *Mycoplasmas* is growing, particularly in immunodeficiency virus-infected individuals. With this in mind, our results show the circulation of these agents in free-roaming canine and feline from Luanda, Angola. Circulation of these agents in uncontrolled and highly mobile animal populations such as synanthropic free-roaming dogs and cats is likely to sustain the transmission in the animal populations due to their close interaction and to the inexistence of ectoparasite elimination programs. The complex interactions between these bacteria, their reservoir hosts and vectors, as well as the broad vector range that include a variety of arthropods, sustain the possibility for interspecies transmission with a high impact on those immunosuppressed individuals.

Further research is needed, including a larger number of free-roaming animals, from other cities and provinces of the country, as well as potential vector ticks and fleas, aiming at better characterizing and controlling vector-borne diseases in Angola.

## 4. Materials and Methods

### 4.1. Animal Recruitment and Data Collection

Between 2018 and 2019 blood samples were collected from apparently healthy, free-roaming cats (*n* = 66) and dogs (*n* = 19) from several parishes of Luanda, Angola, that were submitted to spaying/neutering in a local veterinary clinic. All animals were free-roaming and crossbreed and were included in an animal population control program. Dogs’ and cats’ age was categorized as young (<2 years), adults (2–7 years), and geriatric (>7 years) according to previous studies [36]. Whole blood samples were collected from each cat and dog into ethylenediaminetetraacetic acid (EDTA) tubes and were frozen at −20 °C until further processing. No clinical information was available from these animals. This study was approved by the organism responsible for animal welfare (ORBEA), ICBAS-UP, Portugal, as complying with the Portuguese legislation for the protection of animals (Law no. 2880/2015 and Decree-Law no. 113/2013).

### 4.2. Nucleic Acid Extraction

Total genomic DNA was extracted from 400 μL of EDTA-blood samples using a commercial kit (GRS Pure DNA Kit, Grisp, Porto, Portugal), according to the manufacturer’s instructions. The DNA was eluted in 100 μL elution buffer and stored at −20 °C until use. A negative control (PBS) was used in parallel with the extraction of each set of samples.

### 4.3. PCR Amplification

A total of 85 genomic DNA samples were initially screened for *Bartonella* spp. using a broad-spectrum nested-PCR assay targeting a 16S-23S rDNA intergenic spacer region (ITS), as previously described [58]. Samples were also processed using a PCR protocol based on the amplification of a partial sequence of the 16S rRNA gene of feline hemotropic mycoplasmas [59]. All samples were subjected to a second PCR protocol carried out using specific CMt primers [60]. Subsequently, to obtain a longer *Mycoplasma* sequence and for a better molecular assessment of infections in cats and dogs, a third conventional PCR was performed for the previously obtained PCR positive results, using the universal 16S rRNA gene [61]. The sequences of the primers employed and PCR protocols are shown in Table 1. For all reactions, a total of 5 μL of genomic DNA was added to 5.6 μL KAPA Taq DNA Polymerase mix (KAPA Biosystems, Woburn, MA, USA), 12.4 μL of deionized sterile water and 1 μL (10 μM) of the primers in a 25.0 μL final volume of the reaction mixture. The reactions were carried out in an automatic DNA thermal cycler 100 (Bio-Rad, Feldkirchen, Germany), including negative and positive controls. The PCR amplification products were visualized by Xpert green (Grisp, Porto, Portugal) fluorescence after electrophoresis in a 2% agarose gel at 175 V for 45 min.

All *Bartonella*-positive and *Mycoplasma*-positive amplicons obtained were sequenced for genetic characterization. Amplicons were purified with a GRS PCR and Gel Band Purification Kit (Grisp, Porto, Portugal), and sequencing was performed for both strands of PCR products by the Sanger method, using the respective primers of different target genes.

Sequences were manually corrected using the BioEdit Sequence Alignment Editor v 7.1.9 software package, version 2.1 (Ibis Biosciences, Carlsbad, CA, USA), and further analysis was performed by comparison with the sequences available in the NCBI (GenBank) nucleotide database (http://blast.ncbi.nlm.nih.gov/Blast, accessed on 12 February 2021) [62,63].

## 5. Conclusions

Despite the limited sample size, to our knowledge this is the first report of *Bartonella henselae* infections in stray cats and of hemotropic mycoplasmas in stray cats and dogs from Angola. Population and vector control could contribute to reducing the likelihood of animal-to-animal and animal-to-human transmission. As these infectious agents have a broad vector range that includes both ticks and fleas, transmission between animals and humans is a possibility that deserves further attention. Particular focus should be given to immunosuppressed individuals.

Little is known about the *Bartonella* and hemoplasma prevalence in African countries. These results raise alert for human infection by these infectious pathogens and physicians should consider these agents as possible causes of unexplained fevers in tropical and subtropical African areas.

## Figures and Tables

**Table 1:** Primers and protocols used for the amplification of *Bartonella* spp and feline hemoplasmas gene.

Agents	Target Gene	Primer Primers (5′–3′)	bp	PCR Conditions	Ref
*Bartonella* spp	ITS	P-bhenfa: TCTTCGTTTCTCTTTCTTCA P-benr1: CAAGCGCGCGCTCTAACC	186/168	95 °C, 3 m; 35 cycles (94 °C, 15 s; 48 °C, 30 s; 72 °C, 30 s); 72 °C, 5 m	[43]
*Bartonella* spp	ITS (n-PCR)	N-bhenf1a: GATGATCCCAAGCCTTCTGGC N-bhenr: AACCAACTGAGCTACAAGCC	152/134	95 °C, 3 m; 35 cycles (94 °C, 15 s; 56 °C, 30 s; 72 °C, 30 s); 72 °C, 5 m	[43]
CMhm, Mhf, CMt	16S rRNA	F: ACGAAAGTCTGATGGAGCAATA R: ACGCCCAATAAATCCGRATAAT	170/193	94 °C, 1 m; 35 cycles (94 °C, 1 m; 65 °C, 1 m; 72 °C, 30 s); 72 °C, 5 m	[44]
CMt	16S rRNA	F: AGAGGCGAAGGCGAAAACT R: CTACAACGCCGAAACACAAA	138	95 °C, 2 m; 35 cycles (95 °C, 10 s; 58 °C, 30 s; 72 °C, 30 s); 72 °C 5 m	[45]
Hemotropic mycoplasmas	16S rRNA	F: ATACGGCCCATATTCCTACG R: TGCTCCACCACTTGTTCA	595/618	95 °C, 3 m; 35 cycles (95 °C, 30 s; 60 °C, 30 s; 72 °C, 30 s); 72 °C 5 m	[46]

Abbreviations: n-PCR, nested PCR: CMhm, *Candidatus* Mycoplasma haemominutum; Mhf, *Mycoplasma haemofelis*; CMt, *Candidatus* Mycoplasma turicensis, m, minute, s, seconds.

## Data Availability

Raw data were generated at the Virology Lab of the Faculty of Pharmacy of the University of Porto. Derived data supporting the findings of this study are available from the corresponding author [P.F.B.] on request.
